# Stimulation of allergen-loaded macrophages by TLR9-ligand potentiates IL-10-mediated suppression of allergic airway inflammation in mice

**DOI:** 10.1186/1465-9921-5-21

**Published:** 2004-11-11

**Authors:** Joost LM Vissers, Betty CAM van Esch, Prescilla V Jeurink, Gerard A Hofman, Antoon JM van Oosterhout

**Affiliations:** 1 Department of Pharmacology and Pathophysiology, Faculty of Pharmaceutical Sciences, Utrecht University, Sorbonnelaan 16, 3584 CA Utrecht, The Netherlands; 2 Lab. Allergology & Pulmonary Diseases, Dept. Pathology & Lab. Medicine, Groningen University Hospital, Hanzeplein 1, PO Box 30.001, 9700 RB Groningen, The Netherlands

## Abstract

**Background:**

Previously, we demonstrated that OVA-loaded macrophages (OVA-Mφ) partially suppress OVA-induced airway manifestations of asthma in BALB/c mice. *In vitro *studies showed that OVA-Mφ start to produce IL-10 upon interaction with allergen-specific T cells, which might mediate their immunosuppressive effects. Herein, we examined whether IL-10 is essential for the immunosuppressive effects of OVA-Mφ *in vivo*, and whether *ex vivo *stimulation of the IL-10 production by OVA-Mφ could enhance these effects.

**Methods:**

Peritoneal Mφ were loaded with OVA and stimulated with LPS or immunostimulatory sequence oligodeoxynucleotide (ISS-ODN) *in vitro*. The increase of IL-10 production was examined and, subsequently, *ex vivo *stimulated OVA-Mφ were used to treat (i.v.) OVA-sensitized mice. To further explore whether Mφ-derived IL-10 mediates the immunosuppressive effects, Mφ isolated from IL-10^-/- ^mice were used for treatment.

**Results:**

We found that stimulation with LPS or ISS-ODN highly increased the IL-10 production by OVA-Mφ (2.5-fold and 4.5-fold increase, respectively). ISS-ODN stimulation of OVA-Mφ significantly potentiated the suppressive effects on allergic airway inflammation. Compared to sham-treatment, ISS-ODN-stimulated OVA-Mφ suppressed the airway eosinophilia by 85% (vs. 30% by unstimulated OVA-Mφ), IL-5 levels in bronchoalveolar lavage fluid by 80% (vs. 50%) and serum OVA-specific IgE levels by 60% (vs. 30%). Importantly, IL-10^-/-^Mφ that were loaded with OVA and stimulated with ISS-ODN *ex vivo*, failed to suppress OVA-induced airway inflammation.

**Conclusions:**

These results demonstrate that Mφ-derived IL-10 mediates anti-inflammatory responses in a mouse model of allergic asthma, which both can be potentiated by stimulation with ISS-ODN.

## Background

Chronic asthma is driven and maintained by the persistence of a subset of chronically activated memory T lymphocytes. The development of allergen-specific CD4^+ ^T-helper 2 (Th2) immunoresponses is responsible for the cellular and molecular events underlying the initiation and progression of allergic asthma [1,2]. The Th2 lymphocyte, therefore, is potentially an important target cell for therapy in allergic asthma.

Dendritic cells (DC) are well defined as antigen presenting cells (APC) able to initiate and regulate T cell responses [3]. Besides skewing T-cell responses into Th1 or Th2 responses [4], DC have been shown to mediate the induction of antigen-specific regulatory T (Treg) cells, like CD4^+ ^Th3 cells and CD4^+ ^T regulatory 1 (Tr1) cells [5,6]. Macrophages (Mφ), however, can also serve as APC and play a pivotal role in controlling and directing immune responses [7,8]. To exert these functions, Mφ express MHC-II molecules and secrete a variety of mediators. By secreting pro-inflammatory cytokines, such as IL-1, IL-6 and TNF-α, Mφ can trigger immune responses against microbial pathogens [8,9]. Moreover, by releasing IL-12 Mφ can specifically skew immune responses towards Th1 responses [10-12]. Although Mφ favor the induction of Th1 responses [13,14], it has also been demonstrated that Mφ can induce differentiation of Th2 lymphocytes [15,16]. Similar to DC, Mφ are nowadays thought to be capable of suppressing immune responses by secreting anti-inflammatory mediators, such as PGE_2_, TGF-β and IL-10 [7,9,17].

In the lung, alveolar Mφ participate in the maintenance of immunological homeostasis. By secreting pro-inflammatory cytokines and chemokines they direct the recruitment and activation of inflammatory cells, while they also play a key role in dampening immune responses against non-pathogenic antigens [9,18]. Alveolar Mφ have been shown to suppress T-lymphocyte proliferation *in vitro *[19,20] and APC-function of DC *in vitro *and *in vivo *[21]. Additionally, several studies have demonstrated that Mφ induce tolerance against inhaled allergens, likely at the level of allergen-specific T lymphocytes [22-24]. Interestingly, selective elimination of alveolar Mφ potentiated IgE Ab production in response to inhaled allergen, indicating a key role for alveolar Mφ in tolerance against allergen inhalation [25]. Moreover, we [26] and others [12,27] demonstrated that treatment with allergen-loaded Mφ effectively suppresses allergen-induced airway manifestations of asthma.

*In vitro *studies demonstrated that allergen-specific T cells induced IL-10 production by OVA-loaded Mφ (OVA-Mφ), suggesting that the immunosuppressive effects of OVA-Mφ might be mediated by IL-10 [26]. In this study, we investigated whether stimulation with toll like receptor 4 (TLR4)-ligand LPS [28] and the TLR9-ligand immunostimulatory sequence oligodeoxynucleotide (ISS-ODN) [29] increases the IL-10 production by allergen-loaded Mφ and, thereby, can potentiate their immunosuppressive effects. Subsequently, using Mφ isolated from IL-10^-/- ^mice, we examined whether Mφ-derived IL-10 is crucial in the suppression of allergen-induced allergic airway inflammation.

## Methods

### Animals

Animal care and use were performed in accordance with the guidelines of the Dutch Committee of Animal Experiments. Specific pathogen-free (according to the Federation of European Laboratory Animal Science Associations [30]) male BALB/c mice (6 wk old) were purchased from Charles River (Maastricht, The Netherlands). The mice were housed in macrolon cages in a laminar flow cabinet. Breeding pairs of IL10^-/- ^BALB/c mice were kindly provided by DNAX (Palo Alto, CA) and were housed in macrolon cages with filter top. All mice were provided with food and water *ad libitum*.

### Materials

OVA (chicken egg albumin, grade V) and purified LPS from *Escherichia coli *0111:B4 were purchased from Sigma-Aldrich (St. Louis, MO). CpG-containing phosphorothioate ISS-ODN and control phosphorothioate mutated oligodeoxynucleotide were synthesized by Isogen Bioscience BV (Maarsen, The Netherlands). The ISS-ODN used had the sequence 5'-TGACTGTGAA-CGTTCGAGATGA-3' and the mutated-ODN had the sequence 5'-TGACTGTGAA-GGTTAGAGATGA-3' [31].

### Loading and stimulation of Mφ

Peritoneal Mφ were isolated from naïve BALB/c mice as described previously [26]. For *in vitro *experiments, Mφ were plated in triplicate wells of a 96-well round-bottomed plate (Greiner Bio-One GmbH, Kremsmuenster, Austria) at 1 × 10^5 ^Mφ/well in RPMI 1640 enriched with 2% FCS, penicillin/streptomycin (all GIBCO BRL) and 50 μM β-mercaptoethanol (Sigma-Aldrich). Mφ were loaded with 2 mg/mL OVA and stimulated with different concentrations of LPS, ISS-ODN or mutated-ODN, for 20 h at 37°C and 5% CO_2_. Subsequently, supernatants were harvested and the amount of IL-10 was determined using an IL-10-specific sandwich ELISA. Stimulation with 10 μg/mL LPS or 3 μg/mL ISS-ODN triggered the highest IL-10 production by Mφ.

For *in vivo *studies, 1 × 10^7 ^Mφ/mL were loaded with 2 mg/mL OVA and were stimulated with 10 μg/mL LPS or 3 μg/mL ISS-ODN. After incubation for 3 h at 37°C and 5% CO_2_, the Mφ were extensively washed (3 times with 50 mL saline) to remove all residual soluble OVA, LPS, and ISS-ODN.

### Sensitization, treatment and challenge

Mice were sensitized to OVA by active sensitization with 7 i.p. injections of 10 μg OVA in 0.5 mL pyrogen-free saline on alternate days [32]. Treatment was performed 17 days after the last sensitization by administration (i.v.) of 3 × 10^5 ^Mφ in 50 μl saline. As an additional control group, mice were i.v. injected with 50 μL saline (sham treatment). One week after treatment, mice were exposed to OVA (2 mg/mL saline) aerosol challenges for 5 min on 8 consecutive days.

### Determination of OVA-specific IgE levels in serum

Mice were sacrificed and were bled by cardiac puncture. Subsequently, serum was collected and stored at -70°C until analysis. OVA-specific IgE in serum was measured as described [33]. A reference standard was obtained by i.p. immunization of mice with OVA and arbitrarily assigned a value of 1000 experimental units/mL (EU/mL). The detection level of the IgE ELISA was 0.5 U/mL for IgE.

### Analysis of the cellular composition in the bronchoalveolar lavage fluid

Bronchoalveolar lavage (BAL) was performed immediately after bleeding of the mice by lavage of the airways through a tracheal cannula with 1 mL saline containing 2 μg/mL aprotinine (Roche Diagnostics) and 5% BSA (Sigma-Aldrich). Cytokines in the supernatant of this first mL of the BAL fluid (BALF) were determined by ELISA. Subsequently, mice were lavaged 4 times with 1 mL saline. The cells in the BALF were pooled in cold PBS (including those from the first mL) and subsequently differentiated into mononuclear cells (monocytes, Mφ and lymphocytes), eosinophils, and neutrophils as described previously [33].

### Cytokine ELISAs

IL-5, IL-10, IL-12p70 ELISAs (all BD PharMingen) were performed according to the manufacturer's instructions. The detection limit of the IL-5 ELISA was 10 pg/mL, of the IL-10 ELISA 15 pg/mL, and of the IL-12p70 ELISA 62.5 pg/mL.

### Statistical analysis

All data are expressed as mean ± standard error of mean (SEM). Statistical analysis on BALF cell counts was performed using the non-parametric Mann-Whitney *U *test (2-tailed). For ELISA, results were statistical analyzed using a Student's *t *test (2-tailed, homoscedastic). Results were considered statistically significant at the *P *< .05 level.

## Results

### IL-10 production by Mφ is increased by LPS and ISS-ODN

**Figure 1:**
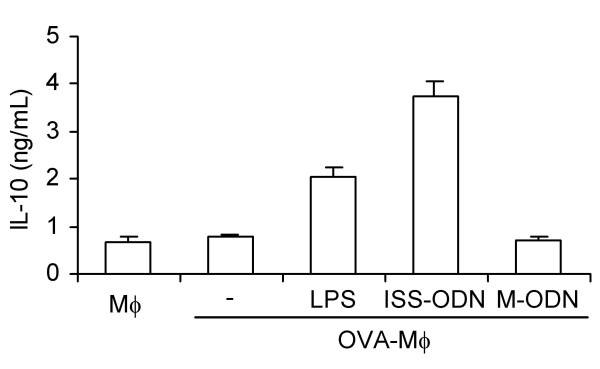
LPS and immunostimulatory sequence oligodeoxynucleotide (ISS-ODN) enhance the IL-10 production by OVA-Mφ. 1 × 10^5^ Mφ/well were loaded with 2 mg/ml OVA and stimulated with either 10 μg/ml LPS or 3 μg/ml ISS-ODN for 20 h. As a control, Mφ were stimulated with mutated-ODN (M-ODN, 3 μg/ml). One of four representative experiments is shown.

*In vitro *studies suggest that the immunosuppressive effects of OVA-Mφ could be mediated by Mφ-derived IL-10 [26]. To further enhance these immunosuppressive effects we attempted to increase the IL-10 levels produced by Mφ. Since Mφ express TLR4 and TLR9 [28,29], we tested whether activation of these receptors (using LPS and ISS-ODN, respectively) would increase the IL-10 production by peritoneal Mφ. As Figure 1 shows, stimulation with LPS or ISS-ODN highly increased the IL-10 production by OVA-Mφ *in vitro*, while control mutated oligodeoxynucleotide did not. The IL-10 levels produced by OVA-Mφ increased 2.5-fold upon stimulation with LPS and 4.5-fold upon stimulation with ISS-ODN. IL-12p70 was not detectable in any of these cultures (data not shown).

### Increased production of IL-10 potentiates the suppressive effects of OVA-Mφ

To examine the *in vivo *effect of the increased production of IL-10 by OVA-Mφ, peritoneal Mφ were isolated and subsequently loaded with OVA and stimulated with LPS (10 μg/mL) or ISS-ODN (3 μg/mL) for 3 h. The stimulated and OVA-loaded Mφ were administered (i.v.) to OVA-sensitized mice.

**Figure 2:**
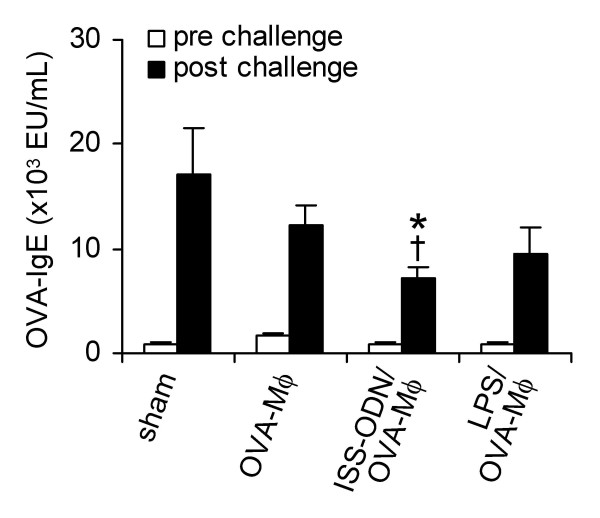
OVA-specific IgE levels in serum are significantly suppressed upon treatment with ISS-ODN-stimulated and OVA-loaded Mφ. OVA-sensitized BALB/c mice were treated (i.v.) with saline (sham), OVA-Mφ, ISS-ODN-stimulated OVA-Mφ (ISS-ODN/OVA-Mφ), or LPS-stimulated OVA-Mφ (LPS/OVA-Mφ). Subsequently, these mice were challenged by OVA inhalation. Serum OVA-specific IgE levels were measured prior to and after challenge. Values are expressed as the mean ± SEM (n = 6 to 8). *P < .05 compared with sham-treated and OVA-challenged mice. ^†^*P *< .05 compared with mice treated with OVA-Mφ and that were OVA-challenged.

In sham-treated mice, OVA-inhalation challenge strongly increased OVA-specific IgE Ab in serum (Figure 2). Treatment with OVA-Mφ that were not stimulated or stimulated with LPS caused no significant suppression in the up-regulation of serum OVA-specific IgE (Figure 2). In contrast, ISS-ODN-stimulated OVA-Mφ significantly suppressed (60%, *P *< .05) the up-regulation of serum OVA-specific IgE (Figure 2). OVA-specific IgG2a levels in serum of sham-treated mice were also increased upon OVA-inhalation challenge. However, these levels were not affected upon treatment with OVA-Mφ or stimulated OVA-Mφ (data not shown).

**Figure 3:**
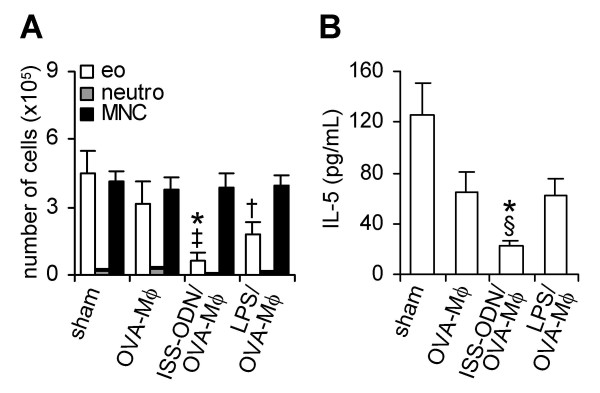
ISS-ODN-stimulated and OVA-loaded Mφ (ISS-ODN/OVA-Mφ) significantly suppress airway eosinophilia and IL-5 levels in the bronchoalveolar lavage fluid. The number of eosinophils (eo), neutrophils (neutro) and mononuclear cells (MNC) in the BALF (A), and IL-5 levels in the BALF (B) after OVA inhalation challenge. Values are expressed as the mean ± SEM (n = 6 to 8). *P < .01 and ^†^*P *< .05 compared with sham-treated mice. ^‡^*P *< .01 and ^§^*P *< .05 compared with mice treated with OVA-Mφ.

The BALF of mice, sensitized and challenged with OVA, contained high numbers of eosinophils (Figure 3A). OVA-Mφ partially suppressed (30%, not significant) the influx of eosinophils into the BALF. *Ex vivo *stimulation of OVA-Mφ with LPS further enhanced the suppression of airway eosinophilia (60%, *P *< .05), compared with sham-treated mice (Figure 3A). OVA-Mφ stimulated with ISS-ODN effectively suppressed the airway eosinophilia. The number of eosinophils in the BALF were significantly (*P *< .01) suppressed by 85% compared to sham-treated mice and by 79% compared to mice treated with OVA-Mφ (Figure 3A).

The BALF of sham-treated mice contained high levels of the Th2 cytokine IL-5 (Figure 3B), that correlates with the numbers of eosinophils. Treatment with OVA-Mφ or LPS-stimulated OVA-Mφ reduced the IL-5 levels in the BALF by 50% (p = 0.07 and p = 0.06, respectively), compared to sham-treated mice (Figure 3B). ISS-ODN-stimulated OVA-Mφ, significantly reduced (*P *< .01) the IL-5 levels in the BALF by 80%, compared to sham-treated mice. IL-10 was not detectable in the BALF of any of the mice (data not shown).

Since ISS-ODN-stimulated OVA-Mφ produced the highest levels of IL-10 and most strongly suppressed OVA-induced airway inflammation, we used these Mφ to further analyze the underlying mechanism of immunosuppression by allergen-loaded Mφ.

### IL-10 produced by OVA-Mφ suppress OVA-induced airway inflammation

To prove that IL-10 produced by OVA-Mφ indeed mediates the observed immunosuppressive effects, we isolated peritoneal Mφ from IL-10^-/- ^BALB/c mice. After loading with OVA and stimulation with ISS-ODN *ex vivo*, the IL-10^-/- ^Mφ were administered (i.v.) to OVA-sensitized BALB/c mice.

**Figure 4:**
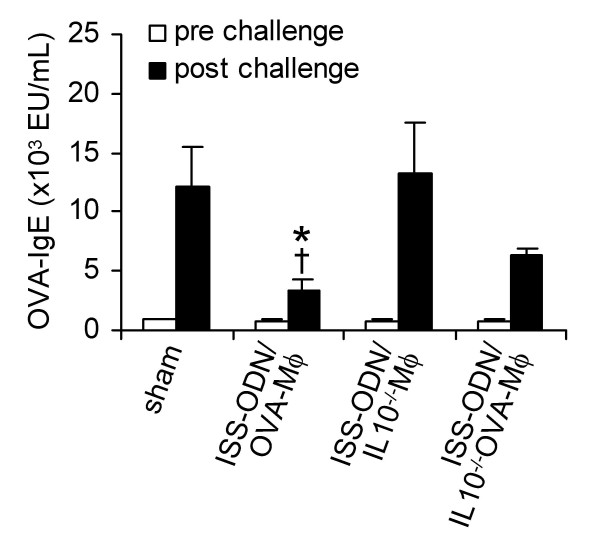
The suppression of OVA-specific IgE in serum by ISS-ODN-stimulated and OVA-loaded Mφ is partially mediated by IL-10. Sensitized mice were treated (i.v.) with saline (sham), ISS-ODN-stimulated OVA-Mφ (ISS-ODN/OVA-Mφ), ISS-ODN-stimulated IL-10^-/- ^Mφ (ISS-ODN/IL10^-/-^Mφ), or ISS-ODN-stimulated IL-10^-/- ^OVA-Mφ (ISS-ODN/IL10^-/-^OVA-Mφ). Serum OVA-specific IgE levels were measured prior to and after OVA challenge. Values are expressed as the mean ± SEM (n = 6 to 8 per group). *P < .05 compared with sham-treated and OVA-challenged mice. ^†^*P *< .05 compared with mice treated with ISS-ODN/IL10^-/-^OVA-Mφ and that were OVA-challenged.

Serum OVA-specific IgE levels of allergic mice that were treated with ISS-ODN-stimulated IL-10^-/- ^Mφ were as high as that of sham-treated mice (Figure 4). However, the up-regulation of serum OVA-specific IgE levels was partially affected by ISS-ODN-stimulated IL-10^-/- ^OVA-Mφ (Figure 4). The serum OVA-specific IgE levels were approximately 50% suppressed compared with unloaded ISS-ODN-stimulated IL-10^-/- ^Mφ. Still, these IgE levels were 45% higher (*P *< .05) than in mice treated with ISS-ODN-stimulated OVA-Mφ.

**Figure 5:**
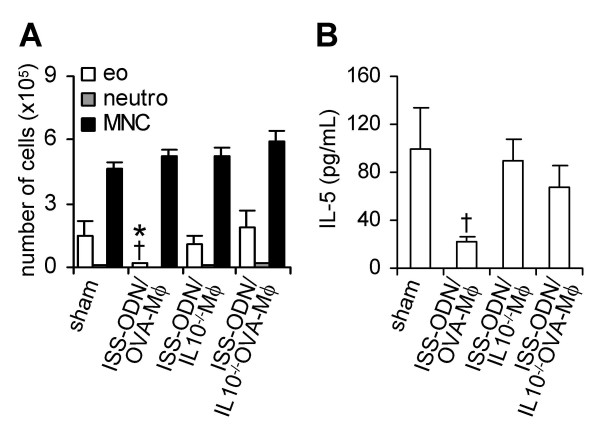
IL-10 is crucial in the suppression of airway eosinophilia and IL-5 levels in the bronchoalveolar lavage fluid by ISS-ODN-stimulated and OVA-loaded Mφ (ISS-ODN/OVA-Mφ). (A) Number of eosinophils (eo), neutrophils (neutro) and mononuclear cells (MNC) in the BALF after OVA challenge. (B) Levels of IL-5 in the BALF after OVA challenge. Values are expressed as the mean ± SEM (n = 6 to 8 per group). *P < .05 compared with sham-treated mice. ^†^*P *< .05 compared with mice treated with ISS-ODN/IL10^-/-^OVA-Mφ.

Importantly, after OVA-inhalation challenge, treatment with ISS-ODN-stimulated IL-10^-/- ^OVA-Mφ did not suppress airway eosinophilia (Figure 5A). Unloaded IL-10^-/- ^Mφ that were stimulated with ISS-ODN had also no effect on airway eosinophilia, while immunotherapy with ISS-ODN-stimulated OVA-Mφ suppressed the influx of eosinophils by 88% (*P *< .05), compared to sham-treated mice (Figure 5A).

The BALF of sham-treated mice, sensitized and challenged with OVA, contained high levels of IL-5 (Figure 5B). Treatment with ISS-ODN-stimulated IL-10^-/- ^OVA-Mφ as well as with ISS-ODN-stimulated IL-10^-/- ^Mφ had no effect on the IL-5 levels in the BALF (Figure 5B). In contrast, these IL-5 levels were significantly reduced by 78% (*P *< .05) upon treatment with ISS-ODN-stimulated OVA-Mφ (Figure 5B).

Together, IL-10 produced by OVA-Mφ mediated the anti-inflammatory effects of allergen-loaded Mφ on allergen-induced airway eosinophilia and IL-5 production.

## Discussion

Previously, we showed that allergen-loaded Mφ partially suppress allergen-induced airway manifestations in mice [26]. Here, we demonstrated that the anti-inflammatory effects of allergen-loaded Mφ are IL-10 dependent and that both the IL-10 production and the immunosuppressive effects can be potentiated by stimulation of ISS-ODN.

Stimulation with ISS-ODN, a ligand for TLR9 [29], readily increased the IL-10-production by peritoneal Mφ that were loaded with OVA. In contrast, these Mφ produced no detectable IL-12p70. We (data not shown) and others [34] confirmed these data using the Mφ-like cell line RAW264.7. In our mouse model of allergic asthma, stimulation of OVA-Mφ with ISS-ODN significantly potentiated their immunosuppressive effects. The suppression of the OVA-induced serum OVA-specific IgE levels, airway eosinophilia and IL-5 levels in the BALF was enhanced. Measuring the enhanced pause (Penh) in response to inhalation of different doses of methacholine (data not shown), we confirmed our previous finding that OVA-Mφ significantly suppressed OVA-induced airway hyperresponsiveness to methacholine [26]. As potentiation of the IL-10 production by Mφ (using LPS or ISS-ODN) did not further suppress the allergen-induced airway hyperresponsiveness (data not shown), it can be speculated that the mechanisms underlying the suppression of airway hyperresponsiveness and of allergic airway inflammation, at least in part, differ. However, we would like to stress that Penh values may not correlate with changes in pulmonary resistance [35].

Compared to ISS-ODN, stimulation with LPS showed an intermediate capacity to enhance the immunosuppressive effects of OVA-Mφ. OVA-Mφ produced higher levels of IL-10 upon stimulation with ISS-ODN compared to stimulation with LPS, suggesting a correlation between the levels of IL-10 produced by the Mφ and the extent of suppression of allergen-induced airway inflammation. As LPS and ISS-ODN trigger signaling via different intracellular pathways [36], we hypothesized that stimulation of Mφ with a combination of LPS and ISS-ODN could further increase the production of IL-10. However, the levels of IL-10 produced by OVA-Mφ stimulated with LPS and ISS-ODN were as high as when stimulated with ISS-ODN only (data not shown). Implying that stimulation of Mφ with ISS-ODN results in maximal production of IL-10.

Using Mφ isolated from IL-10^-/- ^BALB/c mice, we demonstrated that Mφ-derived IL-10 is crucial in the suppression of airway eosinophilia and IL-5 levels in the BALF, while the suppression of serum IgE is partially IL-10 dependent. Although a lack of IL-10 production could up-regulate the MHC class II and B7 expression in Mφ of IL-10^-/- ^mice, as increased IL-10 levels could down-regulate these molecules [37,38], the shift in expression of these molecules will most probably not be the underlying mechanism of suppression of airway eosinophilia and Th2 cytokines, because low levels of MHC class II or B7 itself do not result in suppressive functions. Furthermore, we can not exclude that there are, at present unknown, developmental changes in Mφ derived from IL-10-deficient mice, that may affect their capacity to suppress an allergic inflammatory response. The observation that the suppression of IgE is partially IL-10 independent suggests that the suppression of serum IgE levels is only slightly correlated to Th2-cytokine levels. This is in agreement with the finding that memory IgE responses are inferior mediated by Th2 cytokines [39]. These data indicate that a second, IL-10 independent, suppressive pathway has to be induced by OVA-Mφ that causes a further suppression of serum IgE levels.

Mφ can reside for long period of time in tissue, including the lung [40]. By secreting the immunosuppressive cytokine IL-10 the Mφ could, allergen-independently, suppress allergen-induced airway inflammation. In the past, IL-10 has been shown to suppress allergen-induced airway manifestations of asthma in the mouse [41-43]. However, we found that, after i.v. treatment, the ISS-ODN-stimulated OVA-Mφ specifically migrate to the spleen of OVA-sensitized mice. Subsequently, at this site, an allergen-specific and long-lasting immunosuppressive response is induced (preliminary results by Vissers JLM et al). These results demonstrate that the IL-10 produced by the Mφ is not directly responsible for the suppression of allergic inflammation in the lungs, but that an allergen-specific suppressive T-cell subset is induced in the spleen. This hypothesis is supported by the finding that IL-10 production by OVA-Mφ, upon recognition of OVA-specific T cells *in vitro*, is dependent on MHC class II/TCR interaction [26].

Direct targeting of OVA to alveolar Mφ, for instance by intratracheal treatment with OVA-loaded liposomes, could demonstrate whether Mφ in the lung can directly induce suppression of OVA-induced airway inflammation. However, we (data not shown) and others [44] found that alveolar Mφ from OVA-sensitized mice do not produce IL-10 upon stimulation with LPS or ISS-ODN. Although we were not able to test the suppressive capacity of OVA-loaded alveolar Mφ in our mouse model, targeting of allergens to alveolar Mφ could still be promising to induce immunosuppressive effects in humans, because alveolar Mφ from patients with allergic asthma produce IL-10 [45,46].

In this study and in our previous study [26], we observed no indications for an increased Th1 response upon immunotherapy with OVA-Mφ that could counteract the Th2 response. In contrast, others demonstrated that allergen-loaded Mφ, stimulated with IFN-γ *ex vivo*, promote a switch from Th2 cells to Th1 cells [12,27]. This dissimilarity can mainly be explained by the difference in cytokines which are produced by the Mφ used. IFN-γ-stimulated Mφ produce IL-12 upon allergen-specific interaction with T cells [12], while our Mφ produce IL-10 upon antigen recognition [26]. Likely, IL-12 will favor skewing towards Th1 [4,11], whereas IL-10 will act as a suppressive cytokine. In our model, allergen-loaded Mφ will, most probably, induce Treg cells via secretion of IL-10. Antigen-induced Treg cells are typically induced in microenvironments with APCs presenting antigens and local high levels of IL-10 [6]. This T-cell subset plays a pivotal role in the maintenance of T-cell tolerance against foreign-antigens. They exhibit their suppressive activity by secreting the suppressive cytokine IL-10 (Tr1 cells) or TGF-β (Th3 cells) [47]. By using CD4^+ ^T lymphocytes, *ex vivo *transduced to express IL-10, it was shown that allergen-specific lymphocytes can suppress allergen-induced asthma manifestations via production of IL-10 [43]. Recently, Akbari and colleagues found that pulmonary dendritic cells from mice exposed to respiratory allergen produced IL-10 and, thereby, induced allergen-specific Tr1 cells [5,48]. Furthermore, treatment of mice with killed *Mycobacterium vaccae *induced allergen-specific Treg cells that produced IL-10 and TGF-β [49]. In agreement with our study, these studies indicate a pivotal role for IL-10 in limiting allergen-induced asthma manifestations.

## Conclusions

Here, we demonstrated, in a mouse model of allergic airway inflammation, that treatment with allergen-loaded Mφ suppress asthma manifestations in an IL-10-dependent manner. Importantly, the IL-10 production and anti-inflammatory effects of allergen-loaded Mφ can be potentiated by stimulation with ISS-ODN. Further detailed analysis of the mechanisms underlying this Mφ-based immunotherapy may lead to the development of new strategies to induce tolerance against allergen-specific Th2 responses in allergic diseases, including asthma.

## Authors' contributions

JLMV carried out the allergic model, subsequent analysis, writing and preparation of the manuscript. BCAMvE, PVJ and GAH assisted with the allergic model. AJMvO participated in the direction of the study as well as writing and preparing the manuscript. All authors read and approved the final manuscript.
